# The feasibility of emergency medical technicians performing intermittent high-quality cardiopulmonary resuscitation

**DOI:** 10.7150/ijms.59757

**Published:** 2021-04-29

**Authors:** Chun-Hao Chang, Yi-Ju Hsu, Fang Li, Yuan-Shuo Chan, Ching-Ping Lo, Guan-Jian Peng, Chin-Shan Ho, Chi-Chang Huang

**Affiliations:** 1 Graduate Institute of Sports Science, National Taiwan Sport University, Taoyuan, Taiwan.; 2 Department of Special Education, National Taipei University of Education, Taipei, Taiwan.; 3 College of Exercise and Health Science, National Taiwan Sport University, Taoyuan, Taiwan.; 4 Ching Shuei Emergency Medical Service Team Of 5th Corps, Fire Department, New Taipei City Government, New Taipei City, Taiwan.; 5 Second Special Search and Rescue Branch, Special Search and Rescue Corps, Fire Department, Taoyuan City Government, Taoyuan City, Taiwan.

**Keywords:** CPR, out-of-hospital cardiac arrest, chest compression fraction, fatigue, emergency medical technician

## Abstract

**Background:** Whether intermittent chest compressions have an effect on the quality of CPR is worthy of discussion. The purpose of this study was to investigate differences in the chest compression quality of emergency medical technicians (EMTs) performing cardiopulmonary resuscitation (CPR) with different rest intervals.

**Methods:** Seventy male firefighters with EMT licenses participated in this study. Participants completed body composition measurements and three CPR quality tests, as follows: (1) CPR-uninterrupted for 10 minutes; (2) after 2 days of rest, CPR 10s-intermittent (CPR-10s), for 2 minutes each time and 5 cycles; (3) after another 2 days of rest, CPR 20s-intermittent (CPR-20s), for 2 minutes each time and 5 cycles.

**Results:** Body composition results showed that body mass (BM), body mass index (BMI), upper limb muscle mass (ULMM), core muscle mass (CMM), and upper limb-core muscle mass (UL+CMM) were positively correlated with chest compression depth (CCD) (*p* < 0.05). Analysis of the three different modes of CPR quality analysis indicated significant differences in the chest compression fraction (CCF, F = 6.801, *p* = 0.001), chest compression rebound rate (CCRR, F = 3.919, *p* = 0.021), and ratings of perceived exertion (RPE, F = 23.815, *p* < 0.001). Among the different performance cycles of CPR-10s, significant differences were found in CCF, CCD, CCR (chest compression rate), and RPE (*p* < 0.05). On the other hand, among the different performance cycles of CPR-20s, significant differences were found in CCD, CCR, and RPE (*p* < 0.05). Moreover, the CCF, CCD, and RPE scores of the two tests reached significant differences in specific phases (*p* < 0.05).

**Conclusions:** This study confirmed that the upper limb muscle mass or the weight of the upper body of EMTs is positively correlated with the quality of CPR. In addition, intermittent chest compressions with safe interruption intervals can reduce fatigue caused by long-term chest compressions and maintain better chest compression quality.

## Introduction

Out-of-hospital cardiac arrest (OHCA) is the destructive manifestation of coronary artery disease and commonly leads to high morbidity and mortality 1. Nevertheless, it is recognized that high-quality cardiopulmonary resuscitation (CPR) is a vital determinant of survival following cardiac arrest 2-6 and may achieve the return of spontaneous circulation (ROSC) by providing the required blood flow to preserve significant organ functions 1, 7-11. According to the American Heart Association (AHA) Guidelines for CPR and Emergency Cardiovascular Care, high-quality CPR involves an adequate compression rate and depth, minimized interruptions in chest compressions, full chest rebound, and avoidance of excessive ventilation 12. Of these, effective chest compressions are the most critical aspect of high-quality CPR 2, 11, 13. For manual CPR, the AHA recommends an adult compression depth of at least 5 cm but no greater than 6 cm and a chest compression rate of 100 to 120 beats/min 12, 14.

Adequate chest compressions during CPR are the basis for providing ample blood flow to protect vital organs. However, the quality of manual chest compressions is related to the body weight, body composition, and physical fitness of the rescuer. In a previous study, the relationship between the body weight of rescuers and chest compression quality during CPR was pointed out. The values of oxygen uptake and ratings of perceived exertion (RPE) were dramatically higher in the light group than in the heavy group. In contrast, the heavy group utilized their body weight as a compression force during chest compressions without becoming rapidly fatigued and generally could maintain the ratio of adequate chest compressions 10, 15, 16. Similarly, there is a positive correlation between high BMI and high-quality CPR. Rescuers with higher BMI showed better chest compression performance and less fatigue, while rescuers with relatively lower BMI showed lower chest compression performance 17. Furthermore, according to Kaminska et al. (2018), fat-free mass, trunk muscle mass, left or right arm muscle mass and basal metabolic rate are positively correlated with the depth of manual chest compressions 18.

In clinical settings, rescuers usually have to perform CPR in a kneeling or standing posture with their hands on the patient's sternum while keeping the upper limbs upright 19, 20, which is a very physically exhausting posture and movement. It can be found that a progressive reduction in the percentage of correct chest compressions after 1 minute of chest compressions 15, 21, 22. The reason for the decreasing trend in correct chest compressions could be explained by the rescuer's fatigue, which causes a dramatic decline in the quality of CPR 23. According to statistics from the National Fire Agency of the Ministry of the Interior of Taiwan, the emergency care time (pre-hospital time) after EMTs come into contact with a patient totals 16.64 minutes, and only one EMT cares for the patient in the back of the vehicle on the way to the hospital. Thus, the attending personnel must make a judgment between the quality and time of the first aid. In the case of patients with OHCA, the EMT needs to continue CPR without interruption 24. Therefore, maintaining the quality of CPR is crucial in the long-term pre-hospital stage.

Few studies have evaluated changes in the quality of CPR when rescuers are allowed to rest intermittently during continuous chest compressions. In the study of Min, et al. (2013), the subjects applied three different continuous chest compression methods on a manikin model for 10 min, including continuous chest compressions without a break (CCC), a 10-s break after 100 chest compressions (10/100), and a 10-s break after 200 chest compressions (10/200). The outcomes indicated that the 10/100 method had the highest ratio of adequate chest compressions, followed by the 10/200 and CCC methods, which could be attributed to the rest periods 25. If rescuers are provided with rest breaks during continuous chest compressions, their fatigue will be reduced and the quality of CPR will be improved. The length of the interruptions will affect the survival rate 26, and according to the AHA (2015) Guidelines for CPR and Emergency Cardiovascular Care, the chest compressions should be interrupted as little as possible to maintain the survival rate 12. However, when the manpower is insufficient or the transportation is difficult, there may be no way to perform chest compressions without interruption and thereby maintain the quality of CPR until arrival at the hospital. Perhaps, under the premise of proper care and life monitoring, temporary interruption of chest compressions for rest should be a feasible method.

In view of the above, we assume that resting for a limited interruption time can maintain the CPR compression quality and reduce the fatigue caused by long-term chest compressions. It has also been proposed by previous researchers that the interruption time of chest compression should not exceed 20 seconds 14, 27-29. As far as our knowledge is concerned, there should be no research to compare the effects of different chest compression interruption time on the quality of CPR. Therefore, the purposes of the present study were first, to investigate the correlation between the quality of CPR and the body composition of the emergency medical technician (EMT), and second, to evaluate the impacts of different chest compression methods on the quality of CPR.

## Method and Methods

### Study design

This study recruited firefighters who had received relevant professional training so as to examine the correlation between the quality of CPR performance and body composition and to understand the impacts of different methods of chest compression, two with interruption times, on the quality of CPR. Participants performed CPR quality tests of three chest compression methods on the Laerdal^®^ Little Anne QCPR (Laerdal Medical, Stavanger, Norway) manikin. The study procedure was approved by the Institutional Review Board of Fu Jen Catholic University (New Taipei City, Taiwan) (reference number: C108165). Before the experimental tests began, all subjects completed informed consent forms.

### Participants

A total of 70 active male firefighters from different fire departments in northern Taiwan volunteered to participate in the study. In Taiwan, firefighters are responsible for firefighting and ambulance services, so the recruitment of participants is mainly firefighters. All participants needed to have Emergency Medical Technician-Intermediate (EMT-2) licenses or higher, as well as experience with actual emergency rescue work and CPR first aid. EMT-2 must complete a 24-hour re-training course each year, and Emergency Medical Technician-Paramedic (EMT-P) must complete a 32-hour re-training course each year, both of which include CPR training. The exclusion criteria were diagnoses of cardiovascular disease, chronic disease, or unsuitability for intense exercise. The anthropometry and body composition characteristics of the participants are listed in Table 1.

### Body composition measurement

Body composition was evaluated by standard procedures 30. Participants' body composition parameters were measured with the InBody^®^ 570 Body Composition Analyzer (Biospace, Inc. Seoul, Korea), for which their ages and heights were entered. Height was measured with an H900 height-measurement scale (NAGATA Scale Co., Ltd. Tainan, Taiwan). The InBody^®^ 570 is a reliable method of estimating body composition, including skeletal muscle mass, percentage of body fat, and resting metabolic rate (RMR), with a multi-frequency bioelectrical impedance analyser 30. During the body composition measurement, all participants wore lightweight sportswear and removed their shoes, socks, and any metal objects.

### CPR quality measurement

All participants completed three CPR quality tests in random order. They were required to rest for at least 2 days between each test, and to prevent muscle fatigue or feelings of tiredness, no additional exercise training or weight-bearing work was allowed during the rest period. Before the test, it is necessary to confirm whether the participant has taken 2 days of rest, including at least one day's holiday, and ask whether the participant feels unwell or fatigued before proceeding to the next stage of the test. For all three tests, the participants followed the chest compression technical specifications stipulated by the 2015 AHA guidelines (compression depth of 50-60 mm, compression rate of 100-120 times/min, no interruptions, and full rebound of the chest) 12, with the only differences being the exclusion or inclusion, and the lengths, of the interruptions. No feedback device was used during the tests.

For the CPR-uninterrupted test, the participants performed chest compressions for 10 minutes, without interruptions. Immediately after the test, participants rated their current degree of fatigue degree on the Borg Rating of Perceived Exertion (RPE) scale.

For the CPR 10s-intermittent (CPR-10s) test, the participants performed 5 sets of continuous chest compressions, each lasting 2 minutes and separated by 10 seconds of rest. Immediately after the test, participants rated their current degree of fatigue degree on the Borg RPE scale.

For the CPR 20s-intermittent (CPR-20s) test, the participants performed 5 sets of continuous chest compressions, each lasting 2 minutes and separated by 20 seconds of rest. Immediately after the test, participants rated their current degree of fatigue degree on the Borg RPE scale.

### Data Analysis

All 70 participants completed the study tests. The data from the InBody^®^ 570 Body Composition Analyzer and Laerdal^®^ Little Anne QCPR Manikin, as well as the ratings of perceived exertion (RPE), were all exported to Microsoft Excel (Microsoft Office 2013 for Windows). The body composition parameters of body mass, body mass index, skeletal muscle mass, percentage of body fat, upper limb muscle mass, core muscle mass, upper limb-core muscle mass, and basal metabolic rate of the InBody^®^ 570 Body Composition Analyzer were used in this study. The data of the Laerdal^®^ Little Anne QCPR Manikin were calculated in the SimPad PLUS device (Laerdal, Stavanger, Norway) with activated SkillReporter software. The chest compression quality parameters of chest compression fraction (CCF), chest compression depth (CCD), chest compression rate (CCR), and chest compression rebound rate (CCRR) were used in this study.

### Statistical Analysis

All data are presented as means ± standard deviation (SD). Pearson's coefficient of determination was used to analyze the correlation between body composition and CPR-uninterrupted CPR quality data. One-way analysis of variance (ANOVA) was used to compare the CPR quality of the different chest compression operations. One-way repeated measures ANOVA was used to investigate differences in CPR quality at each phase of the two intermittent times (CPR-10s and CPR-20s tests), respectively. The paired sample t-test was used to analyze the difference in the quality parameters of chest compressions in each phase of CPR-10s and CPR-20s tests. All statistical analyses were performed in IBM SPSS Statistics version 20 (IBM Corp., New York, NY, USA). The significance level was set to α < 0.05.

## Results

### Correlation between body composition and CPR quality

The correlation results of body composition and CPR-uninterrupted CPR quality are provided in Table 2. It was found that BM, BMI, ULMM, CMM and UL+CMM were positively correlated with CCD (BM: r = 0.315, *p* = 0.008; BMI: r = 0.325, *p* = 0.006; ULMM: r = 0.411, *p* < 0.001; CMM: r = 0.393, *p* = 0.001; UL+CMM: r = 0.398, *p* = 0.001). There were no significant correlations between CPR quality and body fat percentage (PBF) (*p* > 0.05).

### CPR quality of different CC interruption times

The ANOVA results for each CPR quality of the CPR-uninterrupted, CPR-10s, and CPR-20s tests are listed in Table 3. The CCF values CPR-uninterrupted (92.70 ± 9.49%) was significantly lower than CPR-20s (97.08 ± 3.32%) (F = 6.801, *p* = 0.001). The CCRR values CPR-uninterrupted (76.83 ± 30.88%) was significantly lower than CPR-20s (87.41 ± 20.93%) (F = 3.919, *p* = 0.021).The RPE values CPR-uninterrupted (14.96 ± 2.58) were significantly higher than those of CPR-10s (13.00 ± 2.12) and CPR-20s (12.36 ± 2.23) (F = 23.815, *p* < 0.001). However, No significant differences were found in CCD or CCR among the three chest compression methods (*p* > 0.05).

### Comparison of CPR quality between CPR-10s and CPR-20s tests

The changes in the CPR quality over the 5 cycles of chest compressions of the CPR-10s and CPR-20s test are listed in Table 4. For the various CPR quality parameters in the CPR-10s test, significant decreases were found in CCF (96.13 ± 6.04% to 92.97 ± 12.74%, *p* = 0.042), CCD (58.46 ± 2.93 mm to 55.11 ± 5.59 mm, *p* < 0.001), and then CCR (107.96 ± 6.92 beats/min to 110.93 ± 7.74 beats/min, *p* < 0.001) and RPE (10.81 ± 1.70 to 14.73 ± 2.81, *p* < 0.001) with increases in the number of cyclic chest compressions, and no significant difference was found in CCRR (*p* = 0.109). For the various CPR quality parameters in the CPR-20s test, significant decreases were found in CCD (58.80 ± 2.88 mm to 56.69 ± 3.67 mm, *p* = 0.001), and then CCR (107.91 ± 5.82 beats/min to 110.96 ± 7.05 beats/min, *p* < 0.001) and RPE (10.47 ± 2.12 to 14.06 ± 2.81, *p* < 0.001) with increases in the number of cyclic chest compressions, but no significant changes were found in CCF (*p* = 0.429) or CCRR (*p* = 0.455) with increases in the number of cyclic chest compressions.

### The difference in the quality of chest compression between CPR-10s and CPR-20s tests

**Figure 1:**
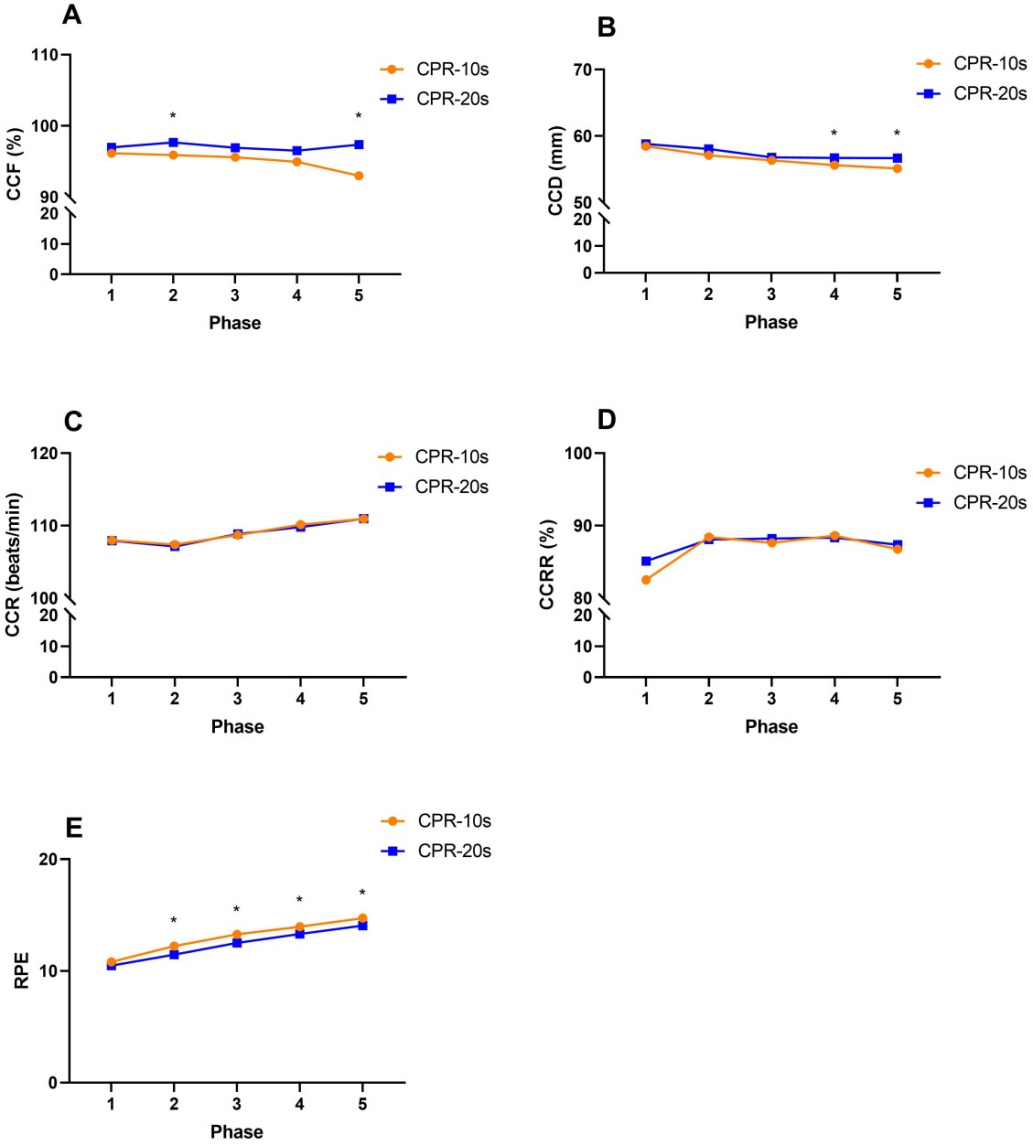
** The difference in the quality of chest compression between CPR-10s and CPR-20s. A**, CCF: chest compression fraction). **B**. CCD: chest compression depth. **C**, CCR: chest compression rate. **D**, CCRR: chest compression rebound rate. **E**, RPE: rating of perceived exertion. *Significantly different from CPR-10s, *p*<0.05.

The difference in the quality of chest compressions between the CPR-10s and CPR-20s tests in each phase is shown in Figure 1. The results showed that phase 2 (0.70 [95% CI, -3.18 to -0.39]; *p* = 0.013) and phase 5 (1.35 [95% CI, -7.06 to -1.68]; *p* = 0.013) of CCF reached a significant difference between the CPR-10s and CPR-20s tests; phase 4 (0.50 [95% CI, -2.08 to -0.09]; p = 0.033) and phase 5 (0.47 [95% CI, -2.51 to -0.62]; *p* = 0.001) of CCD reached a significant difference between the CPR-10s and CPR-20s tests; and phase 2 (0.22 [95% CI, 0.34 to 1.21]; *p* = 0.001), phase 3 (0.22 [95% CI, 0.31 to 1.20]; *p* = 0.001), phase 4 (0.23 [95% CI, 0.18 to 1.11]; *p* = 0.008) and phase 5 (0.25 [95% CI, 0.18 to 1.17]; *p* = 0.009) of RPE achieve significant difference between CPR-10s and CPR-20s test.

## Discussion

High-quality chest compression is an important factor in determining patient survival. We investigated the relationship between the body composition of a firefighter and the quality of CPR, as well as the quality of CPR during chest compressions with different rest periods in this study. The results indicated that, in terms of upper limb muscle mass and core muscle mass, there was a significant positive correlation of body composition with CPR quality. This result is similar to the finding of Ogata, Fujimaru, & Kondo (2015) that, in athletes or workers, physical training of upper body muscle strength is correlated with the quality of CPR or fatigue 31. To reduce the deterioration of CPR quality in firefighters performing chest compressions without interruptions and to prevent hypoxia in the brains of OHCA patients due to excessive CPR interruption times, we assumed that a limited CPR interruption (rest) time could maintain the CPR quality of professional first responders while preventing premature muscle fatigue caused by prolonged chest compressions. We found that only CCRR had no significant difference in quality during the CPR-10s test. However, in the quality of CPR-20s, it can be found that CCF and CCRR did not change significantly with the increase of chest compression operation time. From these results, it appears that the CPR-20s intermittent method can maintain CPR quality for a long time.

The maintenance of high-quality CPR depends on the rescuer's technique and physical fitness. With increases in the first aid time, the rescuer's physical strength will gradually decrease, and this decrease will directly affect the quality of CPR. The results of body composition analysis showed that BM, BMI, ULMM, CMM, and UL+CMM were only positively correlated with CCD in the CPR quality index parameters. This shows that the above body composition parameters of the participants in this study only affect the depth of chest compressions, and do not affect other CPR quality index parameters. The reason may be that the participants already have proficient CPR skills and have good control in CPR operations. Only the body weight and other related parameters will affect the depth of chest compressions. Furthermore, the weight of their body or limbs can be used as the force during chest compression, which in turn affects the depth of compression. A study by Kaminska et al. (2018) pointed out that the basal metabolic rate, fat-free mass (FFM), trunk muscle mass, and left and right arm muscle mass of 100 medical students were positively correlated with chest compression depth. In addition, the chest compression depth can increase by 7.3 mm with every 1 kg increase in arm muscle mass 18. These results strongly indicate that upper limb muscle mass or the weight of the upper body may be one of the factors affecting the quality of CPR.

When EMTs rapidly arrive on the scene for pre-hospital emergency care, the process of transport to the hospital can be viewed as a race of physical fitness against time. The study of Chonde et al. (2020) showed that the median time from arrival to departure of ambulance personnel was 20 min (interquartile range (IQR) 19-20 min), and the time to the hospital was 34 min (IQR 30-45 min), so the duration of pre-hospital CPR was 26 min (IQR 25-40 min) 32. One of the main challenges in transporting OHCA patients to the hospital is the maintenance of high-quality CPR during the transport process, especially in difficult transport conditions 33. Although past studies have found that interruption of chest compressions was a common cause of decreased overall CPR quality, and was associated with a poor prognosis 28, 34-36, it is likely that, if chest compressions can be performed during a long period of transportation or with a manpower shortage under rigorous monitoring of life monitoring equipment, it will be feasible to maintain the CPR quality of the EMT and reduce EMT fatigue through turn-taking, taking rests, or taking rescue breaths within short interruption times. The results of this study showed a trend of improvement in the CCF (*p* = 0.001) and CCRR (*p* = 0.021) scores of the three CPR tests (10-min uninterrupted, CPR-10s, and CPR-20s) with increases in the interruption time. However, in terms of degree of fatigue, the degree of fatigue after the 10-min uninterrupted test was significantly greater than those of the CPR-10s and CPR-20s tests (*p* < 0.001). When the skeletal muscles are activated continuously for a long time, fatigue is prone to appear early, and it will be difficult to maintain the target action. Through temporary rest, it is possible to postpone the early occurrence of fatigue. It should be noted that, because the time of chest compression interruption may affect the survival rate 26, the interruption time should be as short as possible (< 10-20s) to reduce the impact on neurological function, whether traditional or mechanical chest compression is used 14, 27-29. In other words, the interruption time of chest compressions requires careful evaluation and monitoring to ensure the safety of OHCA patients.

The 2015 AHA Guidelines Update for CPR and ECC stipulate the importance and operating specifications of high-quality CPR (CPR administration as early as possible, compression rate of 100-120/min, CCD for adults of 50-60 mm, CCD for infants and children of at least 1/3 of the thickness of the chest, full rebound of the chest, and minimized time and frequency of chest compression interruptions) 12. The results of this study showed that, during increases in the total chest compression time during the CPR-10s test, there were significant decreases in CPR quality in the CPR quality index parameters of CCF, CCD, CCR, and RPE (*p* < 0.05), and only CCRR remained unchanged (*p* > 0.05). In contrast, the results of the CPR-20s test showed that only significant decreases in CPR quality in CCD, CCR and RPE (*p* < 0.05) with increases in chest compression time, while CCF and CCRR did not change significantly (*p* > 0.05). Moreover, the CCF, CCD, and RPE scores of the two tests reached significant differences in specific phases. The CPR-20s method appears to be better able than the CPR-10s method for maintaining CPR quality for extended periods. It can be seen that whether chest compressions are interrupted for rest and the length of the rest period will affect the quality of chest compressions and the degree of fatigue. This finding is similar to that of Zou et al. (2015) 37. When the chest compression rate increases, the chest rebound and compression depth scores worsen, and the onset of fatigue occurs significantly earlier. In the results of this study, only CCRR was not affected by increases in chest compression time.

Many factors affect whether an OHCA patient can achieve ROSC. If each stage can be linked together, the chance of rescuing a person will be greatly improved. It is not difficult to understand that whether OHCA patients receive chest compressions is the focus of the entire CPR process. Since the emergency treatment time (pre-hospital time) after the EMT comes into contact with the patient may be affected by long distances or peak traffic times, or even difficult situations, the time of performing CPR may be extended to dozens of minutes 32, 38. In these dozens of minutes of on-site processing and transportation time, it is critical to perform and maintain high-quality CPR (with ideal compression quality, shortest “hand-off” time, and the best conditions for intervention) to achieve ROSC. According to statistics, less than 11% of patients with OHCA can survive to discharge 39, 40.

In the current situation in Taiwan, for example, non-urban areas are faced with uneven distribution of emergency-related equipment and personnel. In general, ambulances are equipped with one driver with one EMT. If an OHCA patient is transported, the EMT in the patient compartment can only perform CPR as much as possible before arriving at the hospital, and no one is available for turn-taking. Given the insufficiency in the number of EMTs, it is crucial to delay the fatigue caused by performing chest compressions and maintain the quality of CPR. This study has confirmed that an EMT can maintain a high level of CPR quality and reduce fatigue by using an intermittent chest compression method, and interruptions of 20 seconds were found to be superior to interruptions of 10 seconds.

## Limitations

The limitations of this study must be considered. First, the participants were male firefighters with EMT licenses and were qualified to perform the intermittent chest compression tests. The results of the study may not apply to female EMT, or the general public without professional first aid training or hospital personnel with poor physical fitness. Second, the simulator used (Little Anne) has an excessively low impedance, so the results could be different if another Laerdal simulator had been used, such as the Resusci Anne QCPR. Furthermore, this study only investigated the effects and feasibility of intermittent chest compressions on the quality of CPR by using the Manikin and did not directly investigate the survival rates of OHCA patients. It is hoped that the effect of intermittent chest compressions on the survival rate can be further investigated in the future.

## Conclusions

Given the above findings, chest compressions without interruption at the emergency site and during transportation may not allow high-quality CPR to be maintained for a long time. This study has confirmed that the upper body muscle mass of a firefighter with an EMT license is positively correlated with the quality of CPR, and also that the fatigue caused by long-term chest compressions can be reduced, and better CPR quality can be maintained, through intermittent chest compressions. We expect that this method can be applied to first aid training courses and first aid scenes and can become one of the methods of maintaining CPR quality in difficult situations.

## Figures and Tables

**Table 1:** Anthropometry and body composition characteristics of participants

	Mean ± SD	Range
Age (years)	30.00 ± 5.04	21.0-39.0
Height (cm)	175.49 ± 5.03	165.0-186.0
Body mass (kg)	77.79 ± 12.38	54.3-117.9
BMI (kg/m^2^)	25.22 ± 3.61	18.5-35.2
Percentage of body fat (%)	20.21 ± 6.03	9.9-35.3
Basal metabolic rate (kcal/day)	1699.17 ± 153.10	1378-2057
Skeletal muscle mass (kg)	34.97 ± 4.28	25.7-45.0
Upper limb muscle mass (kg)	6.98 ± 1.07	4.5-9.4
Core muscle mass (kg)	27.34 ± 3.15	19.7-34.2
Upper limb+Core muscle mass (kg)	34.32 ± 4.22	24.23-43.6

Mean ± SD, mean value ± standard deviation. BMI, body mass index.

**Table 2:** Pearson correlation (*r*) between body composition and CPR quality

	CCF	CCD	CCR	CCRR	RPE
BM	0.144	0.315*	0.073	-0.059	-0.042
PBF	-0.010	0.066	0.134	0.033	0.060
BMI	0.161	0.325*	0.076	-0.009	-0.109
ULMM	0.217	0.411*	-0.016	-0.102	-0.143
CMM	0.199	0.393*	-0.011	-0.110	-0.128
UL+CMM	0.204	0.398*	-0.012	-0.108	-0.132

*p < 0.05. BM, body mass. PBF, percentage of body fat. BMI, body mass index. ULMM, upper limb muscle mass. CMM, core muscle mass. UL+CMM, upper limb muscle mass plus core muscle mass. CCF, chest compression fraction. CCD, chest compression depth. CCR, chest compression rate. CCRR, chest compression rebound rate. RPE, rating of perceived exertion.

**Table 3:** Comparison of the CPR quality and fatigue with different chest compression interruption times

	CPR-uninterrupted	CPR-10s	CPR-20s	*p*
CCF (%)	92.70 ± 9.49‡	95.10 ± 6.87	97.08 ± 3.32*	0.001
CCD (mm)	56.27 ± 5.03	56.52 ± 4.13	57.41 ± 3.65	0.264
CCR (beats/min)	110.09 ± 9.12	109.01 ± 7.44	108.92 ± 5.77	0.601
CCRR (%)	76.83 ± 30.88‡	86.77 ± 22.21	87.41 ± 20.93*	0.021
RPE	14.96 ± 2.58†‡	13.00 ± 2.12*	12.36 ± 2.23*	< 0.001

*Significantly different from CPR-uninterrupted, *p* < 0.05. † Significantly different from CPR-10s, *p* < 0.05. ‡ Significantly different from CPR-20s, *p* < 0.05. CCF, chest compression fraction. CCD, chest compression depth. CCR, chest compression rate. CCRR, chest compression rebound rate. RPE, rating of perceived exertion.

**Table 4:** Changes in CPR quality and fatigue in different phases of the CPR-10s intermittent method

	Phase 1	Phase 2	Phase 3	Phase 4	Phase 5	*p*
**CPR-10s intermittent method**						
CCF (%)	96.13 ± 6.04#	95.89 ± 5.77#	95.57 ± 7.89#	94.94 ± 8.59#	92.97 ± 12.74*†‡§	0.042
CCD (mm)	58.46 ± 2.93†‡§#	57.11 ± 4.37*‡§#	56.34 ± 4.76*†§#	55.61 ± 5.04*†‡	55.11 ± 5.59*†‡	<0.001
CCR (beats/min)	107.96 ± 6.92§#	107.37 ± 8.09‡§#	108.69 ± 7.96†§#	110.13 ± 8.34*†‡#	110.93 ± 7.74*†‡§	<0.001
CCRR (%)	82.50 ± 28.64	88.40 ± 26.87	87.60 ± 25.30	88.60 ± 23.02	86.74 ± 24.60	0.109
RPE	10.81 ± 1.70†‡§#	12.23 ± 2.07*‡§#	13.27 ± 2.35*†§#	13.96 ± 2.56*†‡#	14.73 ± 2.81*†‡§	<0.001
**CPR-20s intermittent method**						
CCF (%)	96.96 ± 4.20	97.67 ± 2.78	96.91 ± 5.98	96.50 ± 6.56	97.34 ± 3.39	0.429
CCD (mm)	58.80 ± 2.88‡§#	58.06 ± 6.76	56.80 ± 4.37*	56.70 ± 4.06*	56.69 ± 3.67*	0.001
CCR (beats/min)	107.91 ± 5.82§#	107.11 ± 6.01‡§#	108.84 ± 6.07†§#	109.79 ± 6.63*†‡#	110.96 ± 7.05*†‡§	<0.001
CCRR (%)	85.09 ± 26.71	88.07 ± 24.35	88.20 ± 22.22	88.33 ± 22.29	87.34 ± 22.15	0.455
RPE	10.47 ± 2.12†‡§#	11.46 ± 2.19*‡§#	12.51 ± 2.38*†§#	13.31 ± 2.45*†‡#	14.06 ± 2.81*†‡§	<0.001

*Significantly different from Phase 1, *p* < 0.05. † Significantly different from Phase 2, *p* < 0.05. ‡ Significantly different from Phase 3, *p* < 0.05. § Significantly different from Phase 4, *p* < 0.05. # Significantly different from Phase 5, *p* < 0.05.

## Abbreviations

EMTs: emergency medical technicians
CPR: cardiopulmonary resuscitation
CCD: chest compression depth
CCF: chest compression fraction
CCRR: chest compression rebound rate
RPE: ratings of perceived exertion
CCR: chest compression rate
OHCA: out-of-hospital cardiac arrest
ROSC: return of spontaneous circulation
AHA: American Heart Association
EMT-2: Emergency Medical Technician-Intermediate
ULMM: upper limb muscle mass
CMM: core muscle mass
UL+CMM: upper limb + core muscle mass
ANOVA: analysis of variance
PBF: body fat percentage
